# In-Vivo Analysis and Model-Based Prediction of Tensides’ Influence on Drug Absorption

**DOI:** 10.3390/molecules26185602

**Published:** 2021-09-15

**Authors:** Zuzana Vitková, Marián Tárník, Jarmila Pavlovičová, Ján Murgaš, Andrej Babinec, Anton Vitko

**Affiliations:** Institute of Robotics and Cybernetics, Faculty of Electrical Engineering and Information Technology, Slovak University of Technology in Bratislava, 81219 Bratislava, Slovakia; zuzana.vitkova@stuba.sk (Z.V.); jarmila.pavlovicova@stuba.sk (J.P.); jan.murgas@stuba.sk (J.M.); andrej.babinec@stuba.sk (A.B.); anton.vitko@stuba.sk (A.V.)

**Keywords:** tensides, absorption, compartment modelling, in-vivo/model-based analysis

## Abstract

Depending on their concentrations the surface-active substances, tensides (surfactants) can positively or negatively influence the drug absorption, which is widely used in the design of the dosage forms with controlled release. A problem is that the (in-vivo) rate of absorption cannot be directly measured and for that reason, it is frequently substituted by evaluation of the (in-vitro) dissolution. On other hand, a suitably designed pharmacokinetic model can directly predict virtually all pharmacokinetic quantities including both the rate of absorption and fraction of the dose reaching the blood circulation. The paper presents a new approach to the analysis of the rate of drug absorption and shows its superiority over traditional in-vivo approaches. Both the in-vivo analysis and model-based prediction of the tenside (monolaurin of sucrose) influence on the rate of absorption of the drug (sulfathiazole) after instantaneous per-oral administration to rats are discussed. It was found that 0.001% solution of tenside can increase the rate of absorption by cca 50% and a two-fold increase in absolute bioavailability can be reached. Attention is also devoted to the formal requirements laid on the model’s structure and its identifiability. The systematic design, substantiation and validation of a parsimonious predictive model that confirms in-vivo results are presented. The match between in-vivo observations and model-based predictions is demonstrated. The frequently overlooked metaphysics lying behind the compartmental modelling is briefly explained.

## 1. Introduction

Drug delivery systems release drugs either into sites of absorption or directly into the blood circulation. However, every dosage form and every way of administration has its advantages and disadvantages. For instance, though the per-oral administration is the most common and patient compliant route, the drug absorption may be hurdled by some significant influences like the first-pass effect, pH changes, presence of bile salts due to which the permeability of the intestine wall may be negatively influenced. Though many papers deal with modelling of the drug absorption, the models are either of in-vitro or physiological characteristics.

One way to modify the drug absorption from the gastrointestinal tract (GIT) consists in the addition of tensides, which depending on their concentration, may increase or decrease the rate of absorption. It is known that below the critical micellar concentration (CMC), they increase the rate of absorption while above CMC they decrease it [1,2,3]. Parallel to the drug absorption, it also run other processes, e.g., the metabolization and excretion, the evaluation of the rate of absorption by the mere measurement of the blood concentration may be disputable if at all possible. The problem naturally calls for using other ways of absorption evaluation.

Contemporary cybernetics has a decisive impact on the successful solutions of many problems in biology and medicine. There are strong needs to analyse the macro and micro processes running in a live organism. The study of the semantic knowledge extracted from models of in-vivo processes is one of the key topics of bio-cybernetics. Knowledge extracted from observations of living processes and/or historical databases can be relatively easily condensed in the (compartment) models. This helps to reduce a complex biosystem into a finite number of compartments. In this way, the discrepancy between the bio-system complexity and a relatively small number of in-vivo samples that are obtainable from the animal experiments may be significantly minimized.

The problems covered in this paper are partly related to the conditions under which a particular model may replace the in-vivo experiment, but this issue is not explicitly analysed. The impetus for dealing with the problem envisaged in the title came from the need to know the extent to which the tenside influences the rate of absorption and the cumulative drug amount which was actually absorbed from the intestine. Values of these quantities cannot be measured in-vivo, but they can be easily and precisely predicted by an appropriate model.

The compartment model was built upon the results of the in-vivo experiment consisting of the administration of the suspension of sulfathiazole with and without the added tenside (MLS) to rats. The drug without the added tenside was administered first and the corresponding series of concentration samples was recorded. Then, the same process was repeated but with the added tenside. Based on obtained results, a parsimonious structure of the compartmental model was designed, which was subsequently analysed with respect to its parametric identifiability.

The methodology used in the design and identification of the model’s structure can be equally well applied to all per-oral dosage forms, including those with the controlled release or even sophisticated drug delivery systems (DDS).

## 2. In-Vivo Experiment―Material and Method

To secure the uniform dosing, before the in-vivo experiment had been performed, an auxiliary in-vitro experiment with the aim to find whether 0.025% concentration of the tenside (monolaurin of sucrose) is optimal for stabilisation of the suspension. It was found that this concentration is optimal for the prevention of the drug from sedimentation; therefore, that concentration was used in the in-vivo experiment.

The amount of sulfathiazole with the free amino group used in the experiment was assessed by the method of Bratton and Marshall [4]. The related measurements were carried out spectrophotometrically at λ = 545 nm. Water suspensions containing 5% of sulfathiazole with and without MLS were prepared. The dose of 0.5 mL of suspension per 100 g was administered to rats of the strain Wistar with an average weight of 200 g. First, the group of 36 rats was divided into 6 groups. Then, the dose of 50 mg of sulfathiazole was administrated to the first group. After 1 h elapsed, the animals of the first group were anaesthetized and using the heparinized injection syringe with cannula were taken out the samples and the mean value of these 6 samples was calculated. The same procedure was repeated for the second group but the samples were taken after two hours after administration, etc. The mean values are shown in Table 1.

The choice of the suspension as a dosage form was dictated by the unsolvability of sulfathiazole in water.

So as to use the same units, all concentrations were converted into amounts. To this end, we used the relation between the total blood volume (TBV) and the body weight (BW), namely TBV [mL] = 0.06 × BW [g] + 0.77 = 12.77 mL [5]. The obtained results are summarised in Table 1, where c means the drug concentration, m and M are corresponding average drug amounts in the blood after administration of the dose 50 mg without and with the added tenside, respectively.

**Figure 1:**
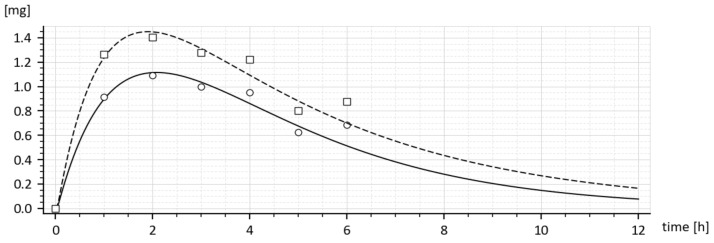
Drug amounts M and m obtained from the in-vivo experiment with (dashed curve) and without (full curve) of the added tenside, respectively.

From observations of the time dependence of the average drug amounts shown in Table 1 and Figure 1, it follows that the maximal average amount was reached 2 h after administration.

The graphs shown in Figure 1 represent the time dependences of the drug amounts in the blood with the tenside (squares) and without the tenside (circles). As can be seen, the highest amount was reached 2 h after administration.

**Figure 2:**
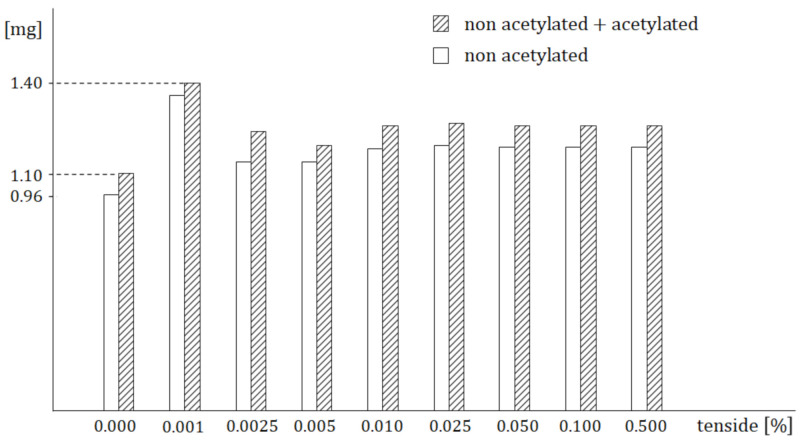
Influence of various concentrations of tenside on the drug amount in the blood.

Analogically, Figure 2 shows that the highest amount (1.4 mg) was reached just for 0.001% solution of MLS and for greater concentrations it remains virtually unchanged. This means that 0.010% concentration of MLS has the strongest influence on drug absorption. The Student’s t-test shows that the maximal statistical significance of differences between the average values of the blood concentrations of the acetylated and non-acetylated sulfathiazole was also recorded for 0.001% of MLS.

Both acetylated and non-acetylated forms of the drug were evaluated. It was found that approximately 8% of the dose acetylates.

## 3. Model-Based Experiment

Well-designed mathematical models of in-vivo processes may be used not only as surrogates of in-vivo experiments but they provide a significantly larger piece of information and deeper knowledge than mere in-vivo experiments. This allegation follows from the generality and flexibility of mathematical modelling. This is the reason that, in this study, the philosophy of compartmental pharmacokinetic modelling was adopted.

### 3.1. Structure of the Model

The specific structure of any compartment model follows from the preliminary analysis of in-vivo measurements. To synthetize a model which would possess sufficient predictive accuracy of the drug amounts in particular parts of the body is a rather difficult task. Practical experience shows that the model with too many tuneable parameters may be rather fragile in the sense that it may accurately predict the system behaviour for one set of the input data but does a poor job for other inputs. Such a model may be too sensitive to even small variations of its structure and/or parameters.

A passable way to overcome some of these problems may be to design a sufficiently parsimonious model structure, which would support the maximal likelihood of the physiologically acceptable hypothesis about the behaviour of the original. According to the principle of parsimony, the model should be maximally descriptive and simple. The model should be designed in accord with the Rule I. of Newton’s Principia, which says: We are to admit no more causes of natural things than those which are both true and sufficient to explain their appearances [6].

**Figure 3:**
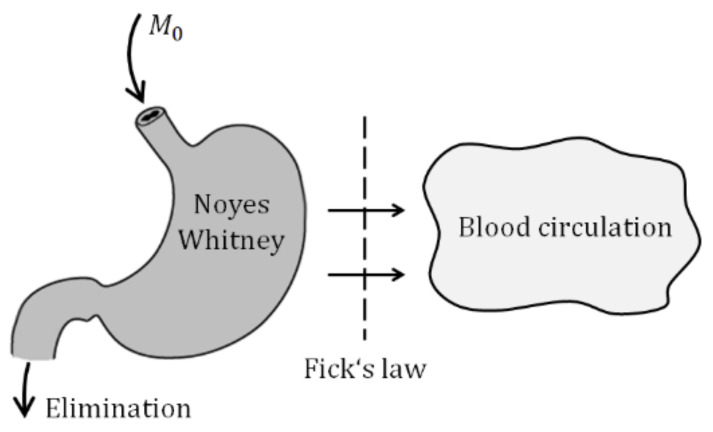
Idea of drug absorption.

A stepping stone of the model derivation is illustrated by two figures and two basic laws describing the drug release and absorption. The solid particles contained in the suspension administrated into the gastrointestinal tract (GIT) must first dissolve and only then they can be absorbed. In parallel with the drug liberation, it is gradually absorbed into the blood circulation and partially eliminated from the body (Figure 3).

The liberation of solid particles in solutions is generally approximated by Noyes–Whitney equation [1].

(1) $$\frac{dM}{dt}=\frac{DA}{h}\left(C_{s}-C\right)$$

The *M* means the mass of particles, *dM/dt* is a rate of liberation, *C_S_* is a saturated concentration at the close vicinity around the particle, *C* is the concentration of the bulk solution, *h* is a thickness of the saturated layer around the particle, *A* is a surface area of the particle, *D* is a diffusion coefficient. As the mass is equal to the product of the volume and concentration, Equation (1) is a first-order differential equation indicating that the liberation obeys the first-order dynamics.

In parallel with the liberation of particles, the drug diffuses through the biological membrane into the blood circulation. The process is commonly described by the Fick law.

(2) $$\frac{dM\left(t\right)}{dt}=-DA\frac{dC\left(x\right)}{dx}$$

The rate of the diffusion *dM(t)/dt* is proportional to the concentration gradient *dC/dx*, which is commonly approximated by the difference of concentrations between the two sides of the biological membrane. Therefore, one can write (2) in the form (3), which says that the diffusion obeys the first-order dynamics as well.

(3) $$\frac{dM\left(t\right)}{dt}=-DA\frac{C_{2}-C_{1}}{d}=+DA\frac{C_{2}+C_{1}}{d}=G\left(\frac{m_{1}}{V_{1}}-\frac{m_{2}}{V_{2}}\right)$$

**Figure 4:**
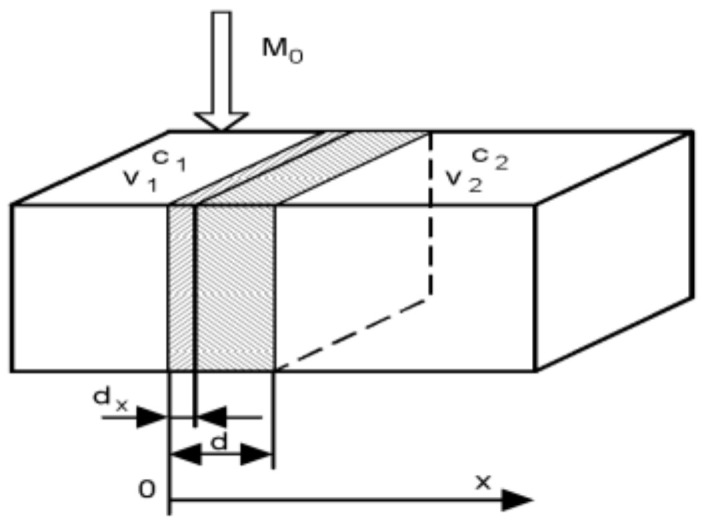
Illustration of the Fick law.

Symbols *m*_1_ and *m*_2_ are drug amounts and *V*_1_, *V*_2_ are volumes of distribution. The constant parameters in (3) are commonly lumped into the membrane permeability *G = DA/d*, which is experimentally determinable. The Equation (3) is illustrated in Figure 4.

Equations (2) and (3) describe two essential sub-processes, namely the drug liberation which is followed by the diffusion through a biologic membrane. These sub-processes drive the drug into motion throughout the body. From the perspective of modelling, Equations (11) and (3) decide about the structure of the future compartment model.

### 3.2. General Properties of Compartment Model

The drug distribution throughout the body is a complex interplay of numerous processes, many of which are still poorly understood. It is commonly modelled by a system of ordinary linear or nonlinear differential equations. The idea behind the compartmental modelling supposes that the living body consists of a few (fictitious) volumes―the compartments. The drug is homogenously distributed inside compartments and flows from one compartment to another. The flows are oriented from the donor compartment with higher concentration to the acceptor compartment with lower concentration. The instantaneous rates of the flows are proportional to the concentrations in donor compartments.

A crucial question is related to the number of compartments. The answer is rather complex from the aspects of both the system theory and physiology. The thing is that the model’s structure must be physiologically substantiated, interpretable, sufficiently general and suitable for the design of a possible control method. According to the principle of parsimony, the number of compartments should be minimal but the model as a whole should adequately describe the corresponding phenomena while avoiding over-interpretations of data. Nevertheless, the system-based analysis suggests universal principles resolving the great majority of many sophisticated problems of the compartmental systems.

Special attention will be devoted to the system identifiability [7]. This property can be elucidated as follows: from the in-vivo measurements, one can obtain nothing more than a series of the time-ordered concentration samples as a response to the administrated dose [8,9,10,11,12]. The in-vivo measurements can provide nothing more than a relationship between the input *u(t) = M*_0_ and the output *y(t)* (samples of concentrations). In other words, the relation between the input (dose administered) and the samples measured at the system output (drug concentrations) defines an input/output (I/O) model, which says nothing about the internal structure of the model. One such I/O model is known as a transfer function. What is worse, the same transfer function corresponds to infinitely many structures (so-called realizations). This fact can cause significant difficulties if one wants to identify values of the model’s parameters―the rate constants of the drug disposition. Therefore, besides the I/O model, a closer look is thrown on the state-space model, also the “internal model”.

### 3.3. State-Space (Internal) Model

The paper uses a linear compartment model with a single input and single-output (SISO) model. It is generally described by the set of linear differential Equations (4). By convention, the matrices are typed by the ordinary upper-case fonts while the lower-case bolt fronts are reserved for vectors. The state vector *x* has *n* components―the number of compartments, and components of *x* are concentrations or amounts of drug in particular compartments.

(4) $$\frac{dx\left(t\right)}{dt}=Ax\left(t\right)+bu\left(t\right)\;y\left(t\right)=c^{T}x\left(t\right)\;x(t_{0})=x_{0}$$

Meanings of denotations:

*x(t)* is a vector of the state variables, e.g., drug amounts or concentrations at the time t

*x(t*_0_*)* is an initial state of the system, i.e., a vector of initial values of the state variables

*u(t)* is an input to the system, e.g., the time profile of the drug amount delivered into the body

*y(t)* is an output from the system, e.g., a drug amount or concentration in the output compartment

*A* is a system matrix―a relation between the state and its time derivative

*b* and *c* are control and observation vectors, respectively

*c^T^* is the transposition of *c*, hence *c^T^* is a row vector

As was said earlier, every compartment model should be physiologically substantiated. The compartment models belong to the category of so-called positive systems, i.e., the systems whose output *y* and all components of the vector *x*, as responses to any positive (nonnegative) input *u* (e.g., the dose administrated) must be positive (nonnegative). That is a natural requirement as to the drug amounts/concentrations, as well as the rates of the drug flows between compartments are always nonnegative. As a consequence, the state trajectory *x(t)* starting at the time *t*_0_ from a nonnegative initial state vector *x(t*_0_*)* must remain consistently nonnegative. It can be shown that this happens if and only if the vectors *b* and *c* are component-wise nonnegative and the matrix A is the so-called Metzler matrix, i.e., a non-zero matrix with nonnegative off-diagonal entries [13,14,15]. Let us note that the requirement of the system positivity significantly hardens the synthesis of possible control algorithms, e.g., the dosing regimes.

So as the state-space model (4) to be a true representative of a compartment model should match the theory of positive and compartment systems [15,16]. In the realm of drug design, this means that the fulfilment of conditions (5) must be checked during the design of the model’s structure.

(5) $$\begin{array}{c} b_{i},c_{i}\;\;\;\;\;\mathrm{Entries of the vectors b and c are non-negative}\\ a_{\mathrm{ii}}\leq 0\;\;\;\;\;\mathrm{Diagonal entries of the matrix A are non-positive}\\ a_{\mathrm{ij}}\geq 0,i\neq j\;\;\;\;\;\mathrm{Off-diagonal entries are non-negative (Metzler matrix)}\\ -a_{\mathrm{ii}}\geq \sum_{l\neq i,l=1}^{n}a_{\mathrm{li}}\;\;\;\;\;\mathrm{Matrix A is column-wise diagonally dominant} \end{array}$$

**Remark** **1.** *Clearly, the drug concentration in an extravascular site of administration is not accessible for measurements. This is one of the reasons why the researchers sometimes evaluate the absorption from the in-vitro 3. Strictly speaking, the (in-vivo) rate of absorption cannot be directly evaluated either from the in-vitro or in-vivo experiment. The only passable way of evaluating the rate of absorption leads through its prediction by a mathematical model.*

**Figure 5:**
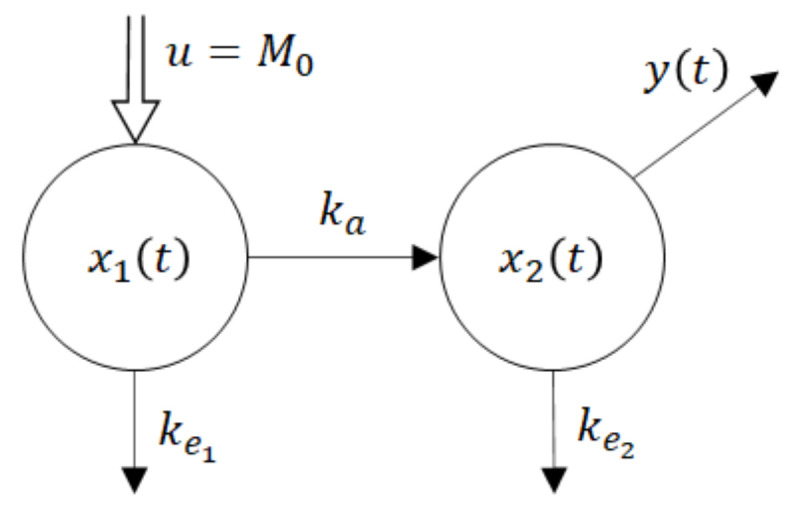
Two-compartment model of per-oral instantaneous administration.

*One of the parsimonious models solving this problem is shown in Figure 5. Obviously, its structure is physiologically acceptable as the drug flows from the site of administration―the GIT into the central compartment―the blood. However, at the same time, this fact is the model’s beauty bug as to the model does not quite correspond to the Ficks law, according to which the rate of the drug flow through the membrane is proportional to the difference between concentrations (amounts) at the opposite sides of the membrane. Namely, in accordance with the law of donor control, the rate of absorption from the GIT equals the product* (*x_1_(t)* × *k_a_*). *However, in the case of the model shown in Figure 5, it means that the rate of absorption does not depend on the drug amount* *x_2_(t)* *in the blood compartment. In the cybernetic parlance, one would say that the rate of absorption “lacks the feedback information” about the current drug amount in the acceptor compartment. As a consequence, the rate of absorption is not influenced by the drug amount* *x_2_(t)*. *Clearly, in reality, such a feedback always exists. No wonder that in some cases the authors approximate the rate of absorption by the exponential* *K* × *exp (−k_a_t),* *which in general is not quite correct, especially for the special drug forms like the transdermal patches, chewing gums, etc.*

*To put things in the right place, in the Appendix A, another compartment model is briefly mentioned, which perfectly matches the law of donor control. However, contrary to the previous case, its physiological correctness may be disputable for a simple reason: Under normal (healthy) conditions the drug does not flow from the blood circulation into the content of the stomach and intestine. Nevertheless, for the purposes of this study, both models are quite good as they generate exactly the same values of the absorption rate constant. The reasons are rather deep, and we omit here the related discussion.*

*End of the remark 1.*

*u* = *M*_0_ is a system input―an instantaneously administered dose

*k*_*e*_1__, *k*_*e*_2__ are rate constants of the drug elimination

*x*_1_*(t)* is a time course of the drug amount in the peripheral compartment―the GIT

*x*_2_*(t)* is a time course of the drug amount in the central compartment—the blood

*y(t)* is a system output―the observed time course of the drug amount

The dynamic behaviour of the compartment model in Figure 5 is described by the state-space model (6). For the sake of notational simplicity, the time dependence of variables is not explicitly expressed.

(6) $$\begin{array}{l} \underset{\dot{x}}{\underbrace{\left(\begin{array}{c} {\dot{x}}_{1}\\ {\dot{x}}_{2} \end{array}\right)}}=\underset{A}{\underbrace{\left[\begin{array}{cc} -\left(k_{a}+k_{e_{1}}\right) & 0\\ k_{a} & -k_{e_{2}} \end{array}\right]}}\underset{x}{\underbrace{\left(\begin{array}{c} x_{1}\\ x_{2} \end{array}\right)}}+\underset{b}{\underbrace{\left(\begin{array}{c} 1\\ 0 \end{array}\right)}}u\\ y=\underset{c^{T}}{\underbrace{\left(0,\;1\right)}}\underset{x}{\underbrace{\left(\begin{array}{c} x_{1}\\ x_{2} \end{array}\right)}}\;\;\;\;\;\;\;\;\;\;\;\;\;\;\;\;\;\;\;\;\;\;\;\;\;\; \end{array}$$

As indicated earlier, an important issue that ought to be examined is identifiability of the matrix A, i.e., the determination of numerical values of its entries from in-vivo samples. However, the crux of the problem lies in the fact that in-vivo samples bear only information about the output *y* as a response to an input *u,* and nothing more. Information about the state variables *x*_1_, *x*_2_ is not included in these samples. The reason is simple: The state variables *x*_1_, *x*_2_ are in general artificial quantities, which may not have a realistic interpretation. Their meanings are defined by the model’s structure which was chosen by the designer. Hence, to resolve the problem of identification of the matrix A one should make a record of an input–output model (I/O model). We decided to use the I/O model in the form of the transfer function *G(s)*, which is the ubiquitous concept in the realm of system analysis. The *G(s)* is defined by the ratio of the image *y(s)* of the output *y(t*) to the image *u(s)* of the input *u(t).* Omitting the detailed explanations, we only declare that the symbol “*s*” is a certain complex variable. The transfer function *G(s)* of the system (4) can be computed in accordance with Expression (7).

(7) $$G\left(s\right)=\frac{y\left(s\right)}{u\left(s\right)}=c^{T}{\left(sI-A\right)}^{-1}b$$

Application of Expression (7) on Equations (6) gives (8).

(8) $$G\left(s\right)=\left(0,1\right){\left[\begin{array}{cc} s+k_{a}+k_{e_{1}} & 0\\ k_{a} & s+k_{e_{2}} \end{array}\right]}^{-1}\left(\begin{array}{c} 1\\ 0 \end{array}\right)$$

After doing requested inversion and multiplications one obtains (9).

(9) $$G\left(s\right)=\frac{y\left(s\right)}{u\left(s\right)}=\frac{k_{a}}{\left(s+k_{a}+k_{e_{1}}\right)\left(s+k_{e_{2}}\right)}$$

Finally, after doing the product in the denominator of (9) one will obtain (10).

(10) $$G\left(s\right)=\frac{k_{a}}{s^{2}+\underset{a_{1}}{\underbrace{\left(k_{a}+k_{e_{1}}+k_{e_{2}}\right)}}s+\underset{a_{0}}{\underbrace{\left(k_{a}k_{e_{2}}+k_{e_{2}}k_{e_{1}}\right)}}}\;=\frac{b_{0}}{s^{2}+a_{1}s+a_{0}}$$

The reason for using different denotations for parameters in (10), namely (*b*_0_, *a*_0_, *a*_1_) and (*k_a_*, *k*_*e*_1__, *k*_*e*_2__) is that while the former were identified (!) from the in-vivo samples, the latter were calculated (!) from already known values of the coefficients *b*_0_, *a*_0_, and *a*_1_). Comparing the homothetic coefficients in the numerator and denominator in (10) the following relations will be obtained:

(11) $$\begin{array}{l} k_{a}=b_{0}\\ k_{a}+k_{e_{1}}+k_{e_{2}}=a_{1}\\ k_{a}k_{e_{2}}+k_{e_{2}}k_{e_{1}}=a_{0} \end{array}$$

Solutions to the Equation (11) for unknown rate constant *k*_12_, *k*_21_ and *k_e_* take the form (12).

(12) $$\begin{array}{l} k_{a}=b_{0}\\ k_{e_{2}}=\frac{a_{1}\pm \sqrt{a_{1}{}^{2}-4a_{0}}}{2}\\ k_{e_{1}}=a_{1}-b_{0}-k_{e_{2}} \end{array}$$

If it would be possible to find unique values of rate constants *k*_*e*_1__, *k*_*e*_2__, *k_a_*, for already known values of the parameters *b*_0_, *a*_0_, *a*_1_, the compartment model (6) would be parametrically identifiable. However, looking at the second expression in (12), it is clear that there exist two possible solutions for *k*_*e*_2__. Hence, the compartment model (6) is not uniquely identifiable.

**Remark** **2.** *The dose u = M_0_ in Figure 5 corresponds to the instantaneous administration of 50 mg of sulfathiazole. Let us recall that the computer implementation of the dose u may be either in the form of the function u(t) = 50 δ(t) mg, where δ(t) means the Dirac unit function [15], or in the form of the initial condition x_1_(0) = 50 mg though the latter alternative may seem to be more natural (because the suspension Though the latter alternative may seem to be more natural (because the suspension was instantly inserted into the stomach), this paper prefers the first one. A reason is that the drug must first transit from the stomach into the intestine where it is dissolved, and only then it can be absorbed. In other words, the drug amount in the GIT―x_1_(t) in (6) cannot instantly jump to the initial value X_1_(0) = 50 mg. Using the initial condition in the role of the input can be fully accepted in the case of an instantaneous intravenous administration but not in the case discussed here. As to the Dirac function δ(t) is defined as an infinitely short and infinitely high impulse with unit surface area, the input u(t) = 50 δ(t) was approximated by a very thin and very high rectangle of the surface area equal to 50 units. In particular, the following dimensions of this rectangle were used: width × height = 0.083333 × 600 m = 50 h·mg.*

*End of remark 2.*

**Figure 6:**
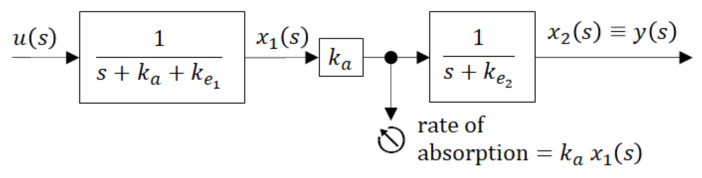
Block scheme with partial transfer functions of the compartment model (6).

To gain better insight into the inner dynamics of the compartment model shown in Figure 5 it may be illustrative to present it by the blocking scheme with partial transfer functions (in rectangles) as shown in Figure 6.

The block scheme shown in Figure 6 is an elegant representation of the compartment model. As a serial connection of partial transfer functions, it distinguishes between the dynamics of the individual compartments and shows the directions of causality. The global transfer function *G(s)* is given by the product of partial transfer functions. It is obviously the same as it is in (9), but it explicitly indicates that the sum (*k_a_* + *k*_*e*_1__) exclusively influences the dynamic behaviour of the 1st compartment, while the dynamics of the 2nd compartment is exclusively influenced by *k*_*e*_2__. The lager is the sum (*k_a_* + *k*_*e*_1__) the more quickly the 1st compartment responds to the input *u(t)*.

Parameters *b*_0_, *a*_0_, *a*_1_ were identified from in-vivo measurements and the following results were obtained: *b*_0_ = 0.030272, *a*_0_ = 0.230137, *a*_1_ = 1.018397. With parameters *b*_0_, *a*_0_, *a*_1_ identified, the rate constants *k*_*a*_, *k*_*e*_1__, *k*_*e*_2__ were calculated in accordance with (12) and two different sets of solutions were obtained:

*k_a_* = 0.030272 h^−1^       *k*_*e*_1__ = 0.649656 h^−1^       *k*_*e*_2__ = 0.338477 h^−1^

or(13)

*k_a_* = 0.030272 h^−1^       *k*_*e*_1__ = 0.308204 h^−1^       *k*_*e*_2__ = 0.679928 h^−1^

Though values of *k_a_* are the same in both sets, values of *k*_*e*_1__ and *k*_*e*_2__ are different. The existence of two different sets of solutions means that model (6) is parametrically un-identifiable. Nevertheless, this fact is not relevant for the purposes of this paper, as information about the rate of absorption is exclusively carried by the absorption rate constant *k_a_*, which was uniquely identified. Keeping in mind that the major route of the drug elimination is renal excretion, in case of need a passable way to determine which of these two sets of solutions is correct can lead through evaluation of the drug amount excreted into the urine. In that case, it would be enough to evaluate the half-time (*t*_1/2_) and then use the known relation *k*_*e*_1__ = 0.693/*t*_1/2_.

### 3.4. Prediction of the Tenside’s Influence on the Rate of Absorption

In Section 2, based on the in-vivo samples without added tensides, the compartment model was designed and parametrically identified allowing determination of the rate of absorption from the intestine into the blood circulation. In this section, the parameters of the same model will be identified, but for the drug with the added tenside.

The in-vivo samples of the drug with the tenside are given in Table 1 (or by the squares in Figure 1). Based on them, the transfer function (10) was identified and the following values were obtained:

*b*_0_ = 0.045815, *a*_0_ = 0.244092, *a*_1_ = 1.247769 (14)

As before, using (12) were computed the following sets of model parameters:

*k_a_* = 0.045815       *k*_*e*_1__ = 0.959041       *k*_*e*_2__ = 0.242913

or (15)

*k_a_* = 0.045815       *k*_*e*_1__ = 0.197097       *k*_*e*_2__ = 1.004856

**Figure 7:**
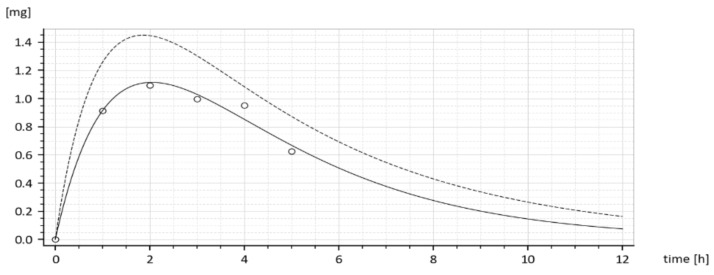
The in-vivo measured drug amounts *x*_2_*(t)* without the added tenside (full curve) and the model predicted amounts with the tenside (dashed curve).

The dashed curve in Figure 7 represents the time course of the drug amount in the blood that was predicted by the model with parameters (15). Obviously, the time course is the same as the upper curve in Figure 1, though these curves were obtained in different ways. Namely, the upper (dashed) curve in Figure 7 was predicted by the model while the upper (dashed) curve in Figure 1 was obtained by the in-vivo measurements. Their sameness proves that both sets of parameters (15) are correct and validates the suggested compartment model.

Let us look at Figure 1. While Figure 1 shows the results of in-vivo measurements, the dotted curve in Figure 7 represents the predicted amounts of the drug with the tenside. The difference between corresponding absorption rate constants *k_a_* is a measure of the strength of the tenside’s influence. As follows from (13) and (15), the 0.001% solution of tenside increased absorption rate constant *k_a_* from 0.030272 h^−1^ to 0.045815 h^−1^*,* that is by 51.34%.

**Figure 8:**
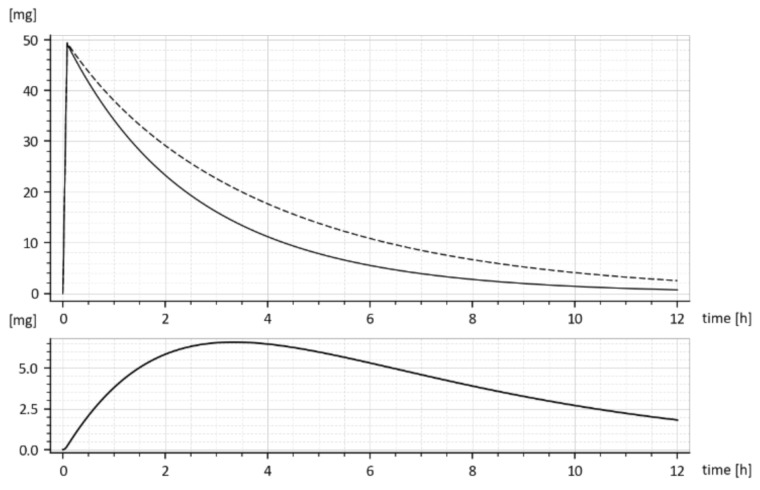
Predicted drug amounts *x*_1_*(t)* in the GIT without (dashed curve) and with the tenside (full curve). Below is the difference (*x*_1without_–*x*_1with_).

The *model predicted* drug amounts it the GIT, i.e., the *x*_1_*(t)*, without and with the tenside are shown in Figure 8. The dashed curve corresponds to parameters (*k_a_* = 0.030272 h^−1^ *k*_*e*_1__ = 0.649656 h^−1^ *k*_*e*_2__ = 0.338477 h^−1^) while the full one corresponds to the increased absorption rate constant *k_a_*, namely (*k_a_* = 0.045815 h^−1^, *k*_*e*_1__ = 0.959041 h^−1^, *k*_*e*_2__ = 0.242913 h^−1^).

One can read, from Figure 8, the free drug amount *x*_1_*(t)* (dashed curve) at a particular time instant, say *t* = 2 h was *cca* 28 mg, but after the addition of the tenside (full curve), it decreased to *cca* 25 mg. The decrease is due to faster outflow from the 1st compartment caused by the increased *k_a_*.

Let us note that *x*_1_*(t)* represents the drug amount in the peripheral compartment (GIT) and for that reason, it cannot be measured in-vivo. It can be only predicted by the compartment model (6). The same goes for the predicted rate of absorption given by Expression (16).

Predicted rate of absorption = *k_a_ x*_1_*(t)*      [mg·h^−1^] (16)

**Figure 9:**
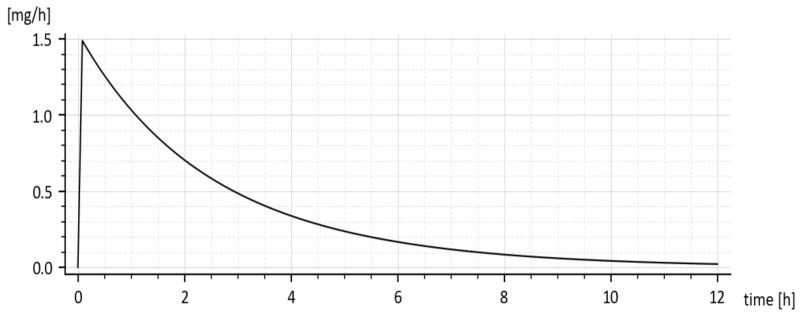
The predicted *rate* of drug absorption (without tenside).

The predicted rate of absorption with added tenside is shown in Figure 9. It copies the global shape of *x*_1_(*t*) without tenside.

The reader is invited to look that the initial onset of the curves in Figure 8 and Figure 9. The curves emanate from the origin (0, 0) and slightly deviate from the vertical axis. It is caused by using the input *u(t) =* 50 δ(*t*)mg rather than the initial condition *x*_1_(0) = 50 mg. The deviation is natural and indicates that the drug amount cannot jump instantly to its initial value.

In the end, it would be worth comparing the predicted results with those obtained in-vivo. As follows from Table 1 and Figure 1, the maximal increase in the drug amount in the blood was caused by the added tenside, which appears at the time *t* = 2 h. The drug in the blood increased from 1.093 to 1.403 mg that is by 28.4%. On other hand, in accordance with Figure 2, (which shows results of the quite independent experiment), the added tenside caused the maximal increase from 1.1 mg to 1.4 mg that is by 27.2%. Taking into account the measurement inaccuracy, the difference between the increase of 28.4% and the increase of 27.2% is virtually insignificant. These findings are another proof of the model’s validity.

#### 3.4.1. Cumulative Eliminated Amounts in Time

**Figure 10:**
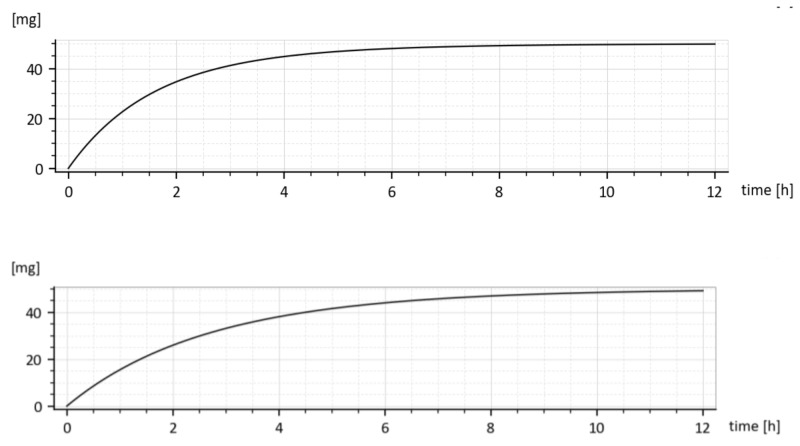
Cumulative amounts of the eliminated drug from the 1st compartment for 1st triplet in (15) (**above**) and the 2nd triplet in (**below**).

As it was already said, the fact that two different values for every elimination constant *k*_*e*_1__ and *k*_*e*_2__ were identified is irrelevant w.r.t. purposes of the paper. This is demonstrated in Figure 10 showing cumulative drug amounts which left the 1st compartment in the time *t*, (given by the integral $\int_{0}^{t}\left[k_{e_{1}}+k_{a}\;\right]x_{1}\left(t\right)dt)\;$for two different parameter sets. Let us consider the parameter sets (15). As shown in Figure 10, both cumulative amounts converge to the administrated dose *M*_0_ = 50 mg, though their shapes are slightly different.

Clearly, the drug leaves the 1st compartment more quickly for the upper triplet of (15) because the sum (*k_a_* + *k*_*e*_1__) is larger than in the lower triplet.

#### 3.4.2. Total Amount of Absorbed Drug and Absolute Bioavailability

The total amount of the absorbed drug is given is by the time integral (17).

(17) $$Total\;amount\;absorbed=\int_{0}^{\infty }k_{a}\;x_{1}\left(t\right)dt\;\;$$

The time dependences *x*_1_**(*t*)** for the drug with and without added tenside are shown in Figure 8. Due to the added tenside, the absorption rate constant *k_a_* increased from 0.030272 h^−1^ to 0.045815 h^−1^, i.e., by 51.34447% and the total absorbed amount (18) increased virtually two-fold, namely from 4.384808 mg to 8.919179 mg.

(18) $$F=\frac{total\;amount\;absorbed}{administered\;dose\;}\;100\%$$

Proportionally to that, the absorbed fraction F of the administered dose *M*_0_, which is nothing else than the absolute bio-availability defined by (19), increased virtually two-fold, namely from 8.76964% to 17.83835%. Hence, the presence of surfactant significantly increases the bioavailability of sulfathiazole from the suspension. Similarly, it was found that the addition of 0.001% solution of the tenside increased the rate constant *k_a_* by cca 51.34%

## 4. Discussion and Conclusions

Tensides strongly influence drug absorption, which is widely used in the design of dosage forms, the ways of the drug administration and the design of dosing regimen. The paper presents both the in-vivo analysis and system-based prediction of the tenside’s (monolaurin of sucrose―MLS) influence on the rate of absorption of sulfathiazole after instantaneous per-oral application to rats. While the in-vivo analysis is able to provide information on the drug concentrations in the blood before and after the addition of the tenside, it says nothing about the amount of the absorbed drug and the rate of absorption. To improve the situation, a maximally possible parsimonious compartment model was designed, able to predict the missing (unmeasurable) quantities.

The mathematical rigour related to the derivation of constraints (5) was avoided but their fulfilment was checked during the design. The designed compartment model was validated by the in-vivo experiment.

Contrary to models of the drug distribution throughout the body in which the input (in this case the per-oral absorption) is considered as an external source, this paper conceives absorption as an integral part of the drug disposition. In other words, the absorption is considered in the context of concurrently running processes rather than a forced input coming from an external independent source. Such design philosophy has opened the door for designing a maximally parsimonious model able to predict unmeasurable quantities.

In the traditional in-vivo analysis that involves the evaluation and subsequent comparison of concentration-time profiles, the quantitative determination of both the amount and rate of absorption, meant as the drug inflow from the site of administration into the blood circulation, cannot be determined. This is because the current concentration (or amount) of the drug in the site of administration―the gastrointestinal tract—is unknown. Therefore, to express the rate of absorption mathematically, some designers approximate it by an in-advance chosen mathematical function, e.g., the exponential *Kexp(−k_a_t)* and then identify parameters *K* and *k_a_*. However, who knows whether the absorption follows the exponential or any other function? This is especially true for special dosage forms, like chewing gums, dragées, dermal patches and the like. On other hand, the presented system-based analysis can directly predict both the rate of absorption and the fraction of the dose which enters the blood circulation.

There were two conducted in-vivo experiments. The first one was necessary for finding such a tenside concentration which would secure that the maximum amount of the drug can be delivered into the blood circulation. It was found that the requested concentration should be equal to 0.001% (Figure 2). All experiments and measurements were carried out for this concentration. At this point, it would be worth mentioning the following: As follows from Figure 2, for concentrations greater than 0.001% the amount of drug rapidly decreased (probably due to the appearance of micelles) and then was kept at a constant value. Due to this, the concentration of 0.001% could be considered to be an approximate value of the CMC of the tenside (C_24_H_44_O_12_) at the temperature of the experiment, i.e., 38 °C. It is an easy task to calculate the molecular weight of the tenside, namely 524.6 g/mole. Keeping this in mind, the tenside concentration 0.001% corresponds to CMC =1/ 524.6 = 1.91 × 10^−3^ mole/l. Note that we neither measured the CMC exactly nor found it in the literature.

The second experiment was aimed at measurements of the complete time course of the drug amount in the blood as a response to the per-oral dose of 50 mg of the drug with and without the added tenside. However, that was all that could be performed. The evaluation of the time course of the rate of absorption was performed on the basis of the synthetized model.

**Figure A1:**
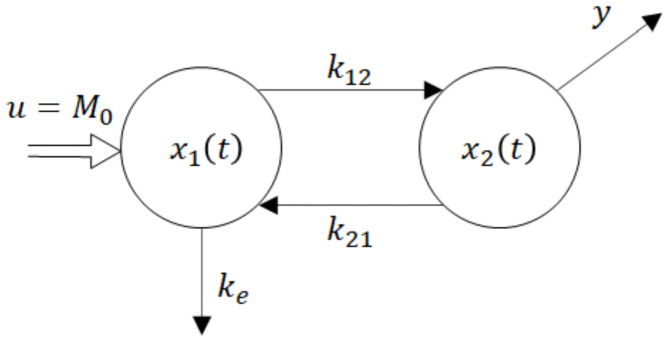
Two-compartment model of instantaneous administration.

As far as the model is considered, a crucial question of compartmental modelling is related to the possibility to uniquely identify values of all model’s parameters. Therefore, there was a need to devote some space to this topic. It was found that the suggested compartment structure yields two possible sets of the model’s parameters. Thus, the model was not uniquely parametrically identifiable though the transfer functions are fully identifiable. In other words, parameters *k_a_* *k*_*e*_1__, and *k*_*e*_2__ cannot be unambiguously determined from already identified values of the parameters *b*_0_, *a*_0_, *a*_1_. The same goes for the model shown in the appendix―see Figure A1. In spite of this, the absorption rate constant *k_a_* was unambiguously determined. In this view, the ambiguous identification of values of *k*_*e*_1__ and *k*_*e*_2__ does not matter. In relation to that, it would be reasonable to note the following: As the major route of the drug elimination is the renal excretion, a passable way to specify which of these two sets of model’s parameters is correct may lead through evaluation of the drug amount in the urine. In particular, it would be enough to evaluate the *half-time* *t*_1/__2_ of the renal excretion and then use the known relation *k*_*e*_1__ = 0.693/*t*_1/2._ However, this would be beyond the scope of the paper.

The proposed system-based approach supports the in-vivo results and conversely, the in-vivo results validate the system-based approach. The compartment model served excellently for simulation and prediction purposes. It completely and exactly predicted the drug fate in the body and the related phenomena. The prediction ability followed from its linearity. The model predicted the concentration profiles for different doses without the need to conduct additional in-vivo experiments.

For the sake of simplicity, the paper does not analyse whether the compartment models shown in Figure 5 and Figure A1 (in the Appendix A) are observable and controllable, but auxiliary analyses certify that this is so. Possessing these properties is important as they secure that, from the known administered dose and the corresponding in-vivo response, it is possible to determine current concentrations (amounts) in all compartments (observability) and that there exists such input to the system (e.g., the time course of the drug delivery) which secures that the desired drug concentrations (amounts) in particular compartments can be reached (controllability). Analysis of these properties is inevitable for the design of drug delivery control, for instance, the dosing regimen. These topics will cover the nearest research activity of the authors

As to the paper works with the averages of six measurements, the results may not quite correspond to the actual processes running in the concrete body. This is especially important when one tries to use the synthetized model for the design of such a dosing regime, which would keep the drug concentration at an optimal level (or at least inside the therapeutic range), in spite of the imprecise or varying parameters. Besides the existence of imprecise parameters, there also exist other sources of uncertainties. To mention only two, it is an un-modelled (neglected) process dynamics (e.g., insufficiently precise approximation of the process dynamics) or some exogenous and endogenous influences acting on the studied subject.

All the mentioned arguments call for adopting such a design philosophy, which would secure that most of the adverse influences will be sufficiently suppressed. Such a design philosophy is known as the “robust design”. A certain degree of robustness follows from the fact that all quantities were processed as mean values of the six measurements. However, in reality, the mean values may vary within a significant range, e.g., 10% of their mean values. To secure reliable predictions in presence of such large variations, the designer should make a record of the advanced methods of the robust design. Therefore, the authors are working on the “robustification” of the designed model. The final aim is to design more reliable dosage regimes, which would respect larger parameter variations. Such an approach will provide more secure information about processes running inside of the body and significantly improve the predictive and explanatory capacity of the model. The metaphysics lying behind the compartmental modelling is briefly explained in Remark1 and Remark2.

In a nutshell, the paper analyses the influence of tenside on drug absorption. To this end, two in-vivo experiments were conducted and their limitations were discussed. To overcome the situations of the in-vivo experiments, two parsimonious models were designed and validated, and the system properties were analysed. It is shown that though the model parameters were not fully identifiable, the rate of drug absorption could be uniquely determined. Due to their linear dynamics, the models are able to easily predict the drug absorption for various doses.

To express the influences of the tenside on pharmacokinetic parameters numerically, the following can be stated: The maximal increase in the drug amount in the systemic circulation (Table 1 and Figure 1) appeared at the time t = 2 h. The amount increased from 1.093 to 1.403 mg; that is, by 28.4%. Similarly, in accordance with Figure 2, which shows the results of the quite independent experiment, the amount increased from 1.1 mg to 1.4 mg, i.e., by 27.2%. Taking into account the measurement accuracy, these results of two independent experiments are virtually the same. The absorption rate constant increased by 51.3% and the absolute bio-availability also increased virtually two-fold, from 8.76964 to 17.83835.

## Figures and Tables

**Table 1:** In-vivo concentrations and amounts of the drug without (m) and with (M) tenside.

T [h]	0	1	2	3	4	5	6
c [mg/mL]	0	0.0715	0.0855	0.0780	0.0735	0.0490	0.0535
m [mg]	0	0.914	1.093	0.997	0.952	0.626	0.684
M [mg]	0	1.262	1.403	1.280	1.222	0.803	0.878

## Data Availability

The in-vivo data supporting the reported results are available from the first author.

## Sample Availability

Samples of the compounds are not available from the authors.

## Appendix A

To secure the correctness of the determination of the absorption rate constant *k_a_*, still another parsimonious compartment model (Figure A1) was analysed.

Meanings of symbols do not require explanations. The dynamic behaviour is described by the state-space Equation (A1). The analysis (not included here) indicates that this model is not fully identifiable as well. Nevertheless, this fact does not preclude the absorption rate constant to be correctly identified.

(A1) $$\begin{array}{c} \underset{\dot{x}}{\underbrace{\left(\begin{array}{c} {\dot{x}}_{1}\\ {\dot{x}}_{2} \end{array}\right)}}=\underset{A}{\underbrace{\left[\begin{array}{cc} -\left(k_{12}+k_{e}\right) & k_{21}\\ k_{12} & -k_{21} \end{array}\right]}}\underset{x}{\underbrace{\left(\begin{array}{c} x_{1}\\ x_{2} \end{array}\right)}}+\underset{b}{\underbrace{\left(\begin{array}{c} 1\\ 0 \end{array}\right)}}u\\ y=\underset{c^{T}}{\underbrace{\left(0,\;1\right)}}\underset{x}{\underbrace{\left(\begin{array}{c} x_{1}\\ x_{2} \end{array}\right)}}\;\;\;\;\;\;\;\;\;\;\;\;\;\;\;\;x_{1}\left(0\right)=administred\;dose\;\;\;\;\;\;\;\;\;\; \end{array}$$

The methodology of the parametric identification of the system without the added tenside was the same as before and the following sets of the parameters’ values were obtained:

(A2) $$({k_{12}}_{\;}=0.030272\;h^{-1},\;k_{21}=0.375903\;h^{-1},\;k_{e}=0.612224\;h^{-1})\;\;\mathrm{or}\;\left({k_{12}}_{\;}=0.030272\;h^{-1},\;k_{21}=0.612224\;h^{-1}{,}^{\;}{k_{e}}_{\;}=0.375903\;h^{-1}\right)$$

Applying the same procedure on the samples containing the tenside (samples M in Table 1) the model (A1) produced the following triples of the parameter values:

(*k*_12_ = 0.045817, *k*_21_ = 0.943229, *k_e_* = 0.258784)

or (A3)

(*k*_12_ = 0.045817, *k*_21_ = 0.258784, *k_e_* = 0.943229)

Clearly, values of *k*_12_, (*which is in essence the absorption rate constant k_a_)* obtained before and after additions of the tenside are exactly the same for both models (6) and (A1). Specifically, *k_a_* = 0.030272 h^−1^ before addition and *k_a_* = 0.045817 after addition of the tenside. These results also confirm that absence of the full parametric identifiability may not preclude the correctness of the results.
